# Human respiratory syncytial virus subgroups A and B outbreak in a kindergarten in Zhejiang Province, China, 2023

**DOI:** 10.3389/fpubh.2024.1368744

**Published:** 2024-02-16

**Authors:** Shelan Liu, Jinren Pan, Yin Chen, Ling Ye, Enfu Chen, Xiaosha Wen, Wenjie Wu, Bing Wu, Xiaoqi Qi, Ta-Chien Chan, Wanwan Sun, Zhao Yu, Tongjie Zhang, Jianbo Yan, Jianmin Jiang

**Affiliations:** ^1^Department of Infectious Diseases, Zhejiang Provincial Centre for Disease Control and Prevention, Hangzhou, China; ^2^Department of Microbiology, Zhejiang Provincial Center for Disease Control and Prevention, Hangzhou, China; ^3^Department of Infectious Diseases, Daishan Country Centre for Disease Control and Prevention, Zhoushan, China; ^4^Chinese Field Epidemiology Training Program, China Centre for Disease Control and Prevention, Beijing, China; ^5^Department of Infectious Diseases, Zhoushan Municipal Centre for Disease Control and Prevention, Zhoushan, China; ^6^Department of Microbiology, Zhoushan Municipal Center for Disease Control and Prevention, Zhoushan, China; ^7^Research Center for Humanities and Social Sciences, Academia Sinica, Taipei, Taiwan; ^8^Zhejiang Provincial Centre for Disease Control and Prevention, Hangzhou, China; ^9^Key Lab of Vaccine, Prevention and Control of Infectious Disease of Zhejiang Province, Hangzhou, China

**Keywords:** human respiratory syncytial virus (HRSV) genotype A, HRSV genotype B, outbreak, family cluster, attack rate, kindergarten

## Abstract

**Background:**

In May–June 2023, an unprecedented outbreak of human respiratory syncytial virus (HRSV) infections occurred in a kindergarten, Zhejiang Province, China. National, provincial, and local public health officials investigated the cause of the outbreak and instituted actions to control its spread.

**Methods:**

We interviewed patients with the respiratory symptoms by questionnaire. Respiratory samples were screened for six respiratory pathogens by real-time quantitative polymerase chain reaction (RT-PCR). The confirmed cases were further sequenced of G gene to confirm the HRSV genotype. A phylogenetic tree was reconstructed by maximum likelihood method.

**Results:**

Of the 103 children in the kindergarten, 45 were classified as suspected cases, and 25 cases were confirmed by RT-PCR. All confirmed cases were identified from half of classes. 36% (9/25) were admitted to hospital, none died. The attack rate was 53.19%. The median ages of suspected and confirmed cases were 32.7 months and 35.8 months, respectively. Nine of 27 confirmed cases lived in one community. Only two-family clusters among 88 household contacts were HRSV positive. A total of 18 of the G gene were obtained from the confirmed cases. Phylogenetic analyses revealed that 16 of the sequences belonged to the HRSV B/BA9 genotype, and the other 2 sequences belonged to the HRSV A/ON1 genotype. The school were closed on June 9 and the outbreak ended on June 15.

**Conclusion:**

These findings suggest the need for an increased awareness of HRSV coinfections outbreak in the kindergarten, when HRSV resurges in the community after COVID-19 pandemic.

## Introduction

Human respiratory syncytial virus (HRSV) is an enveloped virus with a linear negative-sense single-stranded RNA genome belonging to the *genus Orthopneumovirus* within the family *Pneumoviridae* (1). There are two antigenically distinguishable subtypes of HRSV-A and HRSV-B—which can be further classified into 15 genotypes and 30 genotypes, respectively (2). HRSV is by far the major cause of acute lower respiratory tract infections worldwide in infants and children younger than 5 years, with morbidity and mortality risks particularly pronounced in infants under 6 months of age (3, 4).

HRSV is transmitted by direct contact and aerosol droplets, and via contaminated surfaces, initiating viral replication in the respiratory tract after an incubation period of 4–5 days (1). As a highly contagious pathogen, HRSV can cause outbreaks in specific places and populations, such as nursing homes, pediatric wards, neonatal intensive care units, hematopoietic cell transplant center, and long-term care facilities (LTCFs) where people with low immune function gather (5–11). Nosocomial HRSV infection in these high-risk group population often follows a severe course of disease, even resulting in mortality. For instance, Wang et al., reported that an outbreak of severe neonatal pneumonia caused by HSV BA9 in a postpartum care center in Shenyang, China, a total of 20 confirmed cases were identified, including 16 hospitalized neonates(seven admitted to an intensive care unit and nine to general wards) and the other four nursing staff with asymptomatic infections (2). Yohan et al. revealed a nosocomial HRSV outbreak that occurred in March 2019 in a French long-term stay hospital for older adult, with the median age 89 years old and caused two deaths (6). However, HRSV outbreaks in schools and kindergartens have rarely been reported worldwide.

China has had an integrated surveillance system for respiratory infections since 2004. In the largest study on respiratory infections carried out in China, HRSV was the top most frequently detected respiratory virus in children, accounting for 25.7% (12). National surveillance data showed that HRSV-A/B subtypes appear to be temporal, with recirculation occurring periodically (2–3 years natal cycles) and peaking in winter (13). However, in March–April 2023, a resurgence of HRSV and out-of-season peaks was identified (National surveillance report, internal data). The Epidemiological Surveillance Network of Zhejiang Province reported a large HRSV outbreak in a kindergarten in Daishan Country, Zhejiang Province.

The present study describes epidemiological, clinical, and virological investigations of the outbreak and the public health response. To our knowledge, this is the first and largest outbreak caused by mixed genotypes A and B of HRSV in kindergarten in the world. This research will be of interest to health policymakers and planners in the control and prevention of the HRSV outbreak in the future.

## Methods

### Outbreak recognized

On June 8, 2023, Daishan Country Centre for Disease Control and Prevention (CDC) was informed that four children from the same kindergarten were admitted to the pediatric ward of Daishan First People’s Hospital due to fever and acute respiratory infections. In response, the Zhejiang Provincial Centre for Disease Control and Prevention, municipal, and Country CDC initiated an epidemiological investigation to determine the source of origin and scope of the outbreak and to provide control recommendations.

### Kindergarten setting

**Figure 1:**
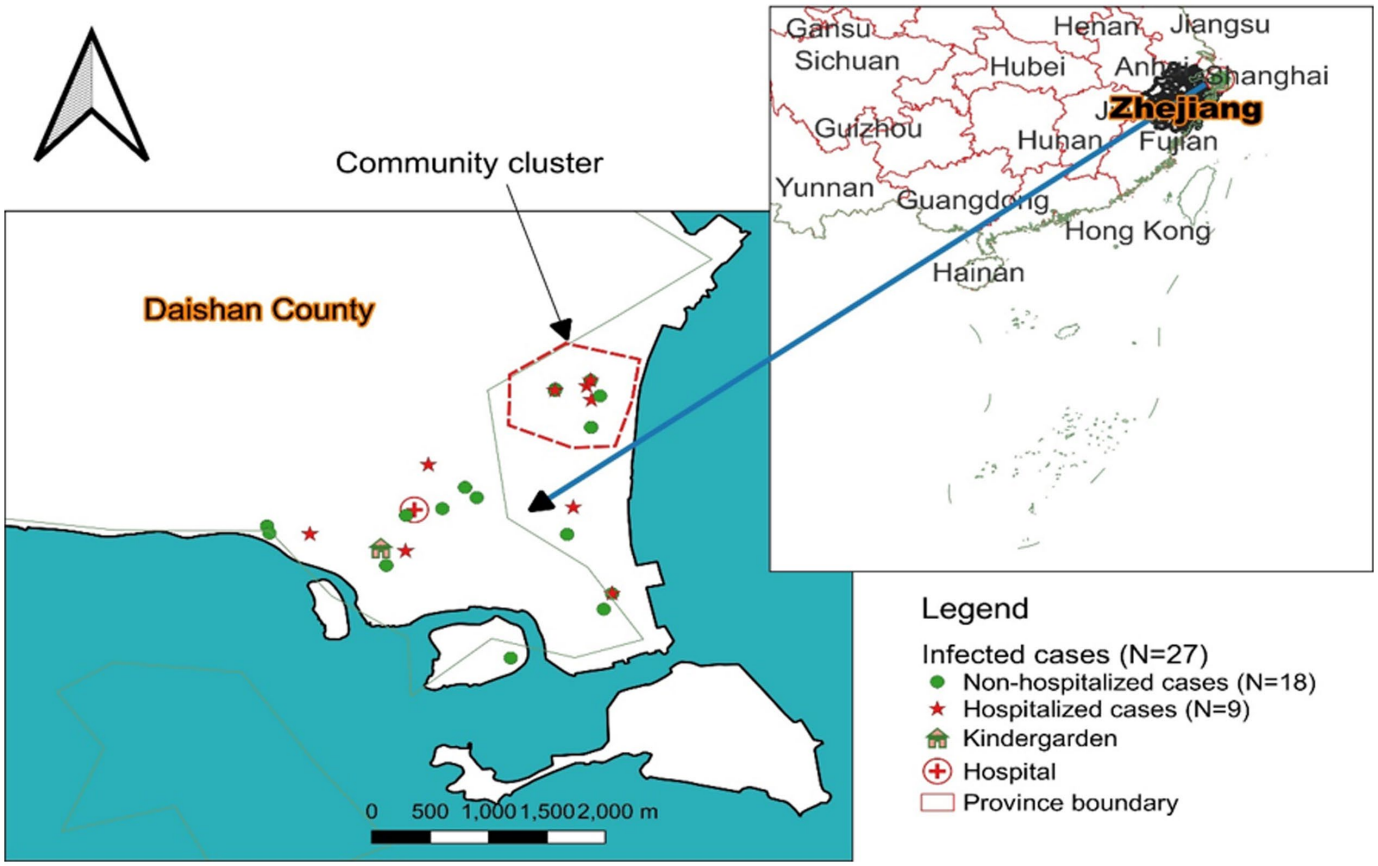
Spatial distribution of 27 confirmed cases caused by human respiratory syncytial virus outbreak in Boji kindergarten, Daishan countryside, Zhoushan city, Zhejiang Province, China during May to June, 2023. (1) House shape sections on the map indicate the outbreak kindergarten; (2) Green dots and five-pointed star indicate HRSV outbreak non-admitted cases and admitted cases respectively; (3) The cross-bonded figure represents the First People’s Hospital of Daishan country where the 9 cases were admitted; (4) Dotted polygons represent clustered cases’ living communities.

Daishan County, located in the northern coastal area of the Zhoushan Islands in Zhejiang Province, China, has approximately 168,000 inhabitants who are mainly farmers and fishermen. There were eight kindergarderns. The Boji kindergarten was in the western north of Gaoting Town of Daishan County (Figure 1), covering a total area of 6,370.2 square metres, of which the building area was 5,200 square metres (Supplementary Figure S1), layout of the first floor of the kindergarten building was seen in Supplementary Figure S2. The kindergarten was opened on September 10, 2021. This kindergarten population comprised 103 pupils and 23 staff members (including one healthcare worker and two cookers). Until the day of this investigation, there were six classes, including four nursery classes (18–19 children/class), one junior class (12 children/class), one senior class (12 children/class) (Figure 1). Each class had two teachers and one nursery teacher. The school day started at 9:00 am and ended at 3–4:00 pm. The kindergarten provided lunch, the food was processed by the on-site canteen, and the food was delivered to each classroom. The teachers had a special staff canteen. The kindergarten children were provided daily with “two meals” and lunch and afternoon snacks. The tables were cleaned before and after meals, and the classrooms were disinfected with a UV lamp for one hour at closing. Diagrammatic sketches of nursery classes 3 and 4 are shown in Supplementary Figures S3, S4. The absentee record by class since May 4 of 2023 is shown in Supplementary Figure S5.

### Case definitions

The case definitions applied followed Chinese HRSV diagnosis guidelines (2), which defined suspect cases of HRSV infection as any child and teacher in kindergarten who, from May 4 (the first suspected case was ill on May 8), developed fever, one acute respiratory symptom (cough, sputum, runny nose, sore throat and other symptoms), and have been excluded from diagnosis of SARS-CoV-2, influenza, other respiratory pathogen’s infections. A confirmed case was defined as a person in whom a real-time reverse-transcriptase-polymerase chain reaction (real-time RT-PCR) of a nasopharyngeal or throat swab sample was positive for HRSV.

### Field investigation

Active case findings were implemented in the kindergarten, and clinical and epidemiological information was obtained using a structured questionnaire that was administered using face-to-face interviews with parents and teachers. The information collected included demographic details, symptoms, recent travel history, and details of out-of-school activities. Information about household and close social contacts and healthcare workers’ contacts was also recorded.

Those who met the case definition were included in the study.

### Specimen collection and transport

During the outbreak period, nasopharyngeal swabs and throat swabs were collected from all children and staff at the kindergarten, their family members, and the hospital. All specimens were kept at 4–8°C and then transported in sterile containers with a cold package (controlled low temperature of 4°C) to Zhoushan CDC for further analysis.

### Real-time RT-PCR and genotyping

The viral nucleic acid was directly extracted from the clinical specimens using a Tianlong nucleic acid extraction kit (Tianlong Biotechnology, Xian, China) according to the manufacturer’s instructions. The samples were screened for human respiratory pathogens, including HRSV virus, SARS-CoV-2, influenza A and B, adenovirus, rhinovirus, and enterovirus, using RT-PCR or PCR with a TaqMan low-density array kit (Thermo Fisher Scientific Inc., Waltham, United States). The HRSV subtypes were further identified using a commercial real-time RT-PCR kit. The second hypervariable region (HVR2) of the G gene of the HRSV was amplified using a one-step reverse transcription PCR kit (Shanghai Zhijiang Biotechnology, China) and the primer pair GPB/F1. The reaction conditions, as well as the purification and sequencing protocols, were as described previously (2).

### Phylogenetic analysis

Sequences were edited with Sequencher 5.0 (GeneCodes, Ann Arbor, MI, United States). Multiple sequence alignments and pairwise distances were determined using the MEGA program (version 5.0; Sudhir Kumar, Arizona State University). Phylogenetic trees were generated using MEGA with the maximum likelihood method. The reliability of the phylogenetic inference was estimated using the bootstrap method with 1,000 replicates. Bootstrap values of >70% are shown.

### Data analysis

Analysis of the epicurve was used to determine the type of outbreak occurring, using the distribution of suspected cases and confirmed cases over time. Attack rates were calculated using the number of confirmed/suspected children as the numerator and the total number of pupils in the school/class as the denominator. Statistical significance was determined using the Chi square test, and the significance level is set to 0.05 to compare with the significance probability value, *p*-value. If the *p*-value is <0.05, it is judged as statistical significance. All reported *p* values are two-tailed.

### Ethical approval

This study was approved by the fourth session of the Ethics Review Committee of Zhejiang Province Centre for Disease Control and Prevention. Written informed consent for the use of clinical specimens was obtained from all patients involved in this study or their guardians. Based on the guidance for Managing Ethical Issues in Infectious Disease Outbreaks. This study did not involve human experimentation; the only human material used in this study was nasopharyngeal swab or throat swab specimens collected from suspected acute respiratory infections (ARI) cases, their family members, and healthcare workers during an outbreak in Daishan Country, Zhoushan city of Zhejiang Province, China, from May to July 2023.

## Results

### Investigation of HRSV outbreak in a kindergarten

The outbreak started from May 18 to June 15, and lasted 28 days. Of the 103 children, 47 were symptomatic, resulting in an overall clinical attack rate of 45.63%. Of the 47 symptomatic cases, 25 (53.19%) were found to be positive for HRSV, resulting in the hospitalization of 9 children. This outbreak had a microbiologically confirmed attack rate of nearly 24.3%. Of the 23 staff members, no one was symptomatic; although 14 required testing, all negative for HRSV. Among the 88 household members of the 25 confirmed cases, 20 were tested, and two symptomatic contacts were positive for HRSV. Both confirmed and suspected cases had no travel histories. All these outbreak cases recovered completely.

#### Area distribution

According to the family living addresses, the 27 confirmed outbreak cases were dispersed in Gaoting Town, Daishan County, with 7 cases living in one community, and the other cases were in different communities (Figure 1).

#### Timeline of infection

**Figure 2:**
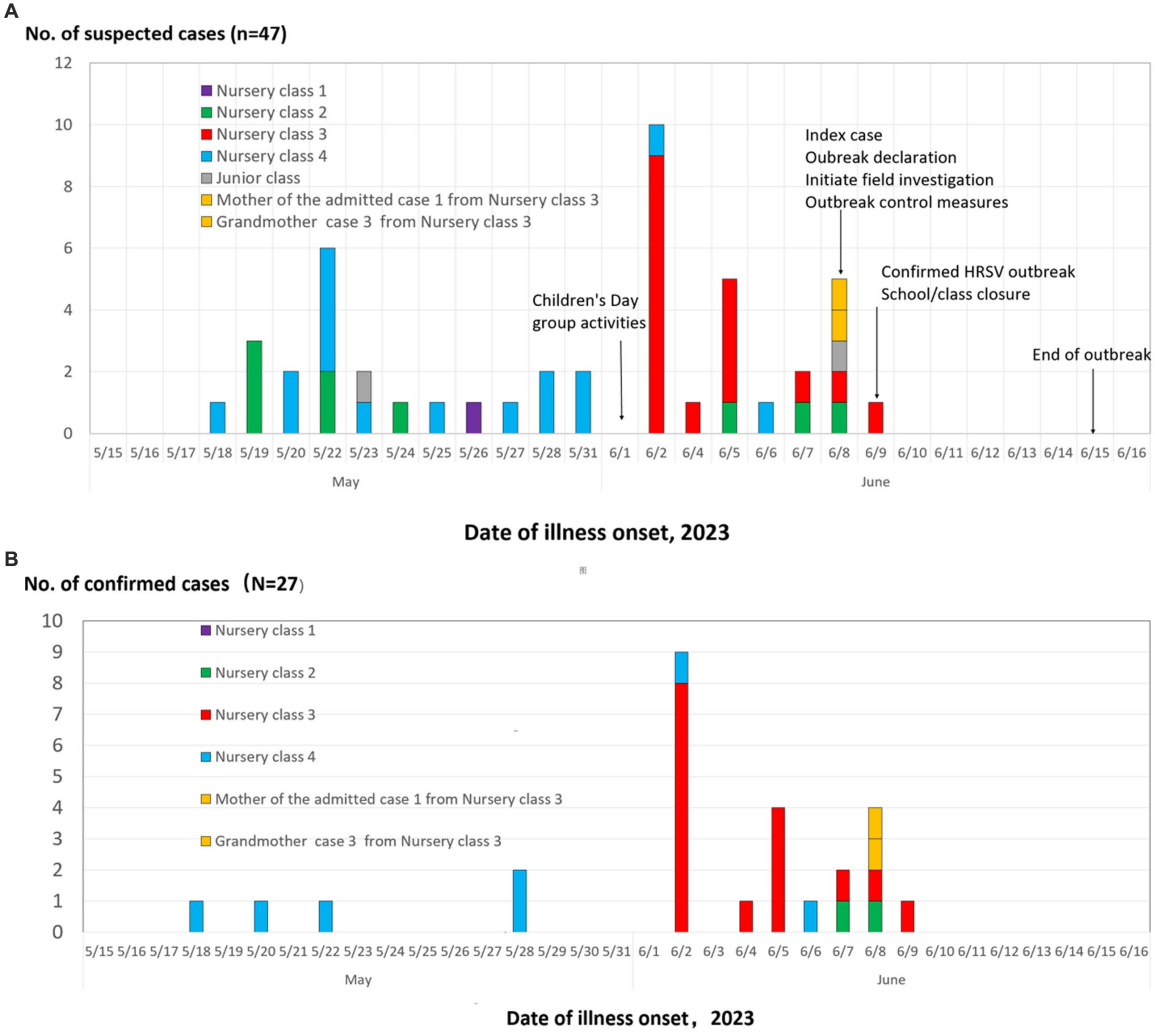
Date of illness onset for suspected and confirmed cases of HRSV among children and household members, by class, in the Boji kindergarten from Daishan countryside, Zhoushan city, Zhejiang Province, China from May to June 2023 (suspected cases = 47; confirmed cases = 27). **(A)** Suspected cases; **(B)** confirmed cases.

The earliest case identified during the investigation was a confirmed admitted case with onset of symptoms on May 18, the outbreak reached its peak on June 2, and no other cases were detected among pupils one week after the last case was reported to be ill on June 9, 2023. The epidemic curve (Figures 2A,B) shows three successive clusters, with the third peak being the highest. The steep decline in cases may have been due to the rapid implementation of control measures, especially the kindergarten/class closure on June 9. The epidemic of class 4 was obviously earlier than that of class 3 (Figures 2A,B).

#### Age distribution

**Figure 3:**
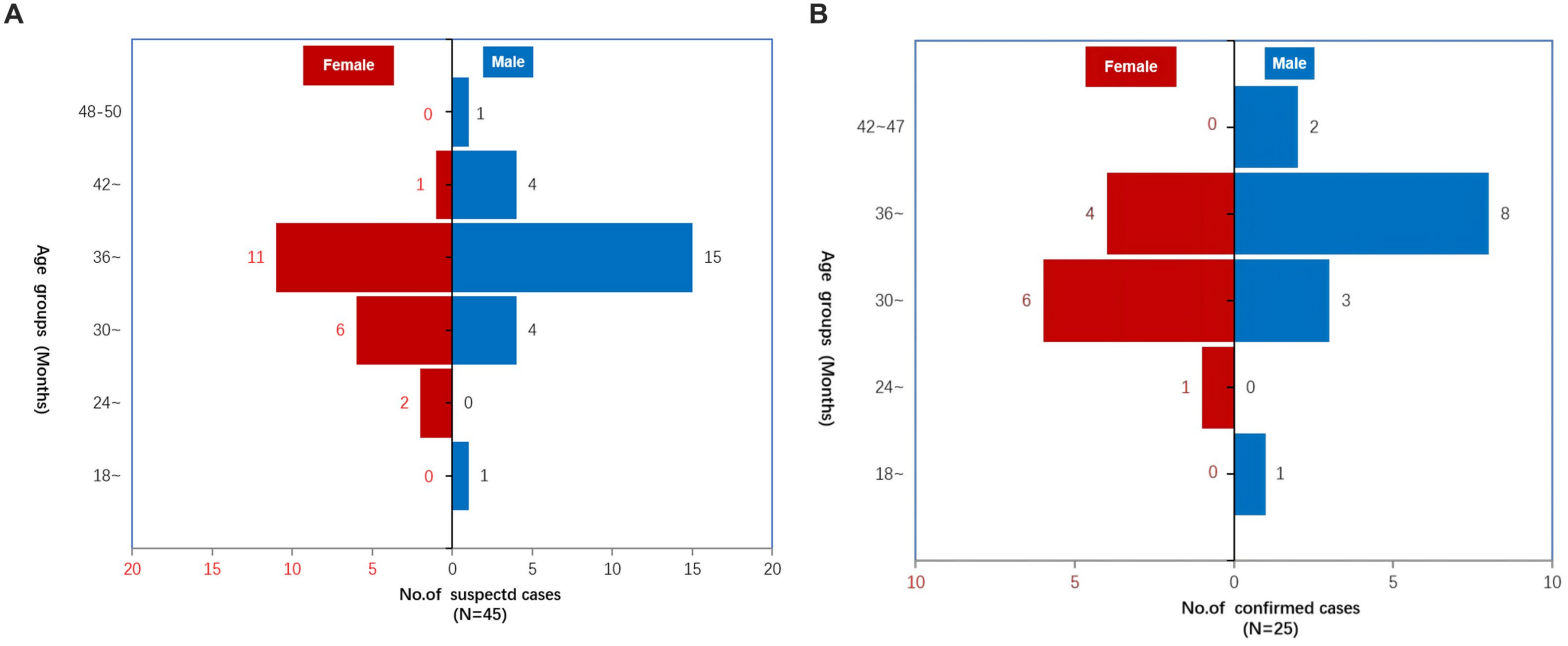
The age distribution of 45 suspected cases and 25 confirmed cases of human respiratory syncytial virus outbreak in the Boji kindergarten from Daishan country, Zhoushan city, Zhejiang Province, China, from May to June 2023. **(A)** Suspected cases; **(B)** confirmed cases.

Among the 45 suspected cases of HRSV, the average age was 32.7 months (range: 18 ~ 50 months) (Figure 3A). Of 25 confirmed child cases, the average age was 35.8 months (Figure 3B), 14 were boys, with a prevalence rate of 25.0%, and 11 were girls, with a prevalence rate of 23.4%, without difference due to gender (*p* = 0.851) (Table 1). All age groups were affected, mainly on children between 30 and 42 months of age, among which the largest number of cases were aged 36–42 months of age (Figure 3B).

**Table 1:** The attack rates for HRSV by grade/age group and sex in 25 HRSV confirmed cases among children in a private kindergarten in Daishan countryside, Zhoushan city, Zhejiang Province, China by date of illness onset, May to June of 2023 (*n* = 25).

Category	No. of cases	No. of children	Attack rate (%)	Attack rate ratio (95% CI)	*p* value
Overall attack rate	25	103	24.3		
Attack rate by sex					
Boys	14	56	25.0	1.091 (0.441, 2.701)	0.851
Girls	11	47	23.4	Reference	
Attack rate by class (age range in months)					
Nursery class 1 (29–45)	0	18	0	0 (0, +∞)	0.998
Nursery class 2 (38–43)	2	18	11.1	Reference	
Nursery class 3 (29–42)	16	19	84.2	42.667 (6.263, 290.649)	<0.001
Nursery class 4 (23–43)	7	18	38.9	5.091 (0.886, 29.265)	0.068
Junior class (42–56)	0	18	0	0 (0, +∞)	0.998
Senior class (59–88)	0	12	0	0 (0, +∞)	0.999

#### Class distribution

This study demonstrated substantial differences in the distribution of cases between classes, or year groups. Half of the classes (3/6) in the kindergarten were affected by the outbreak. The 25 cases were mainly distributed in nursery class 3 (16 cases), nursery class 4 (7 cases), and nursery class 2 (2 cases). Attack rates by grade ranged from 0 to 84.2%. The attack rate was significantly higher (*p* = 0.0014, using two-tailed Chi square test) among nursery class 3 (84.2%) than among children in nursery class 4 (38.9%) and nursery class 2 (11.1%) (Table 1). The highest attack rate was seen in the nursery class 3 (89.5%) and 4 (88.9%) among the suspected cases (Supplementary Table S1).

#### Clinical manifestations and severity

Among the 25 confirmed children’s cases and two family members, 16 children cases and 2 households presented with mild and self-limiting illness, acute respiratory symptoms, and fever (Supplementary Table S2). The initial symptoms were fever, accounting for 81.5% (22/27), with the highest temperature being 39.7°C. The most frequent symptoms were cough (24/27, 88.9%), with productive cough presented by 17 cases (63.0%), while running nose (3/27, 11.1%) and nasal obstruction (2/27, 7.4%) were less commonly reported. None of the cases reported gastrointestinal symptoms, such as vomiting or diarrhoea, and no complications or deaths were reported (Supplementary Table S3).

Nine patients (33.33%, 9/27) were hospitalized for persistent fever, eight of whom developed acute bronchitis (Table 2). Except for one child who was preterm and low weight, the others were healthy and full-term babies. The median age of the hospitalized patients was 2.3 years old, ranging from 1 year to 3 years, and they comprised 5 boys and 4 girls. The median time from illness onset to be confirmed was 2 days (0–4 days). The average hospital stay was 6.4 days (5–9 days) (Table 2). Four cases were hospitalized at the time of the investigation; both were tested for genotype B positive. Four cases from nursery class 4 were coinfected with HRSV and *Mycoplasma pneumoniae.* One two-year-old girl from nursery class 4 was confirmed to have HRSV and SARS-CoV-2 coinfection (Table 2). Among nine admitted cases received symptomatic treatment (such as, relieving cough, relieving asthma and reducing fever, etc.), three of them were treated with antibiotics, six of them were treated with budesonide aerosol inhalation. Finally, all nine cases were discharged and recovered completely.

**Table 2:** Characteristics of nine severe cases of human respiratory syncytial virus infection in patients hospitalized in Daishan First People’s Hospital, Zhejiang Province, China from May to June 2023.

Case no.	Class name	Age (Years)	Gender (M/F)	Weigh of birth (Kg)	Weeks of birth	Onset date	Median day from onset to be admitted	Median day from onset to be confirmed	Hospital stays (Day)	Sample type		*Rt-PCR or PCR*						Diagnosis
											HRSV	Genotype of HRSV	Sars-CoV-2	Influenza	Adenovirus	Rhinovirus	*Mycoplasma pneumoniae*	
1	Nursery class 3	3	M	3	38	6/3	3	3	6	Nasopharyngeal swab	+	B	−	−	−	−	−	Acute bronchitis
2	Nursery class 3	2	F	3.1	39	6/2	3	3	7	Nasopharyngeal swab	+	B	−	−	−	−	−	Acute bronchitis
3	Nursery class 3	3	M	2.5	36	6/3	3	3	7	Nasopharyngeal swab	+	Unknown	−	−	−	−	−	Pneumonia; Allergic rhinitis
4	Nursery class 3	2	F	3.05	38	6/5	0	0	6	Nasopharyngeal swab	+	Unknown	−	−	−	−	−	Acute bronchitis
5	Nursery class 4	1	M	3.85	38	5/28	1	1	6	Nasopharyngeal swab	+	Unknown	−	−	−	−	+	Acute bronchitis
6	Nursery class 4	3	M	3.2	39	6/5	4	4	5	Nasopharyngeal swab	+	Unknown	−	−	−	+	+	Acute bronchitis
7	Nursery class 4	2	F	3.15	38	6/5	3	3	6	Nasopharyngeal swab	+	Unknown	+	−	−	−	+	Acute bronchitis; Acute laryngitis, COVID-19
8	Nursery class 4	2	F	3.35	38	5/23	1	1	9	Nasopharyngeal swab	+	Unknown	−	−	−	−	−	Acute upper respiratory tract infection
9	Nursery class 4	3	M	3.35	38	5/28	2	2	6	Nasopharyngeal swab	+	Unknown	−	−	−	−	+	Acute bronchitis

M, male; F, Female; +means positive; −means negative.

PCR, polymerase chain reaction; RT-PCR, reverse transcription polymerase chain reaction.

#### Laboratory tests

On June 9, 2023, 91 upper respiratory samples were collected, including 77 pharyngeal swabs from children attending kindergarten and 14 from the staff. The results showed that 16/77 of the children were HRSV positive (12 of genotypes B, 2 of genotypes A, and 2 of unknown genotypes); 14/14 of the staff members were all HRSV negative. We collected 24 environmental samples from the kindergarten (from the beds, tables, toys, doorknobs, toilets, countertops, etc.). All environmental samples were HRSV-negative.

### Family transmission investigation

**Figure 4:**
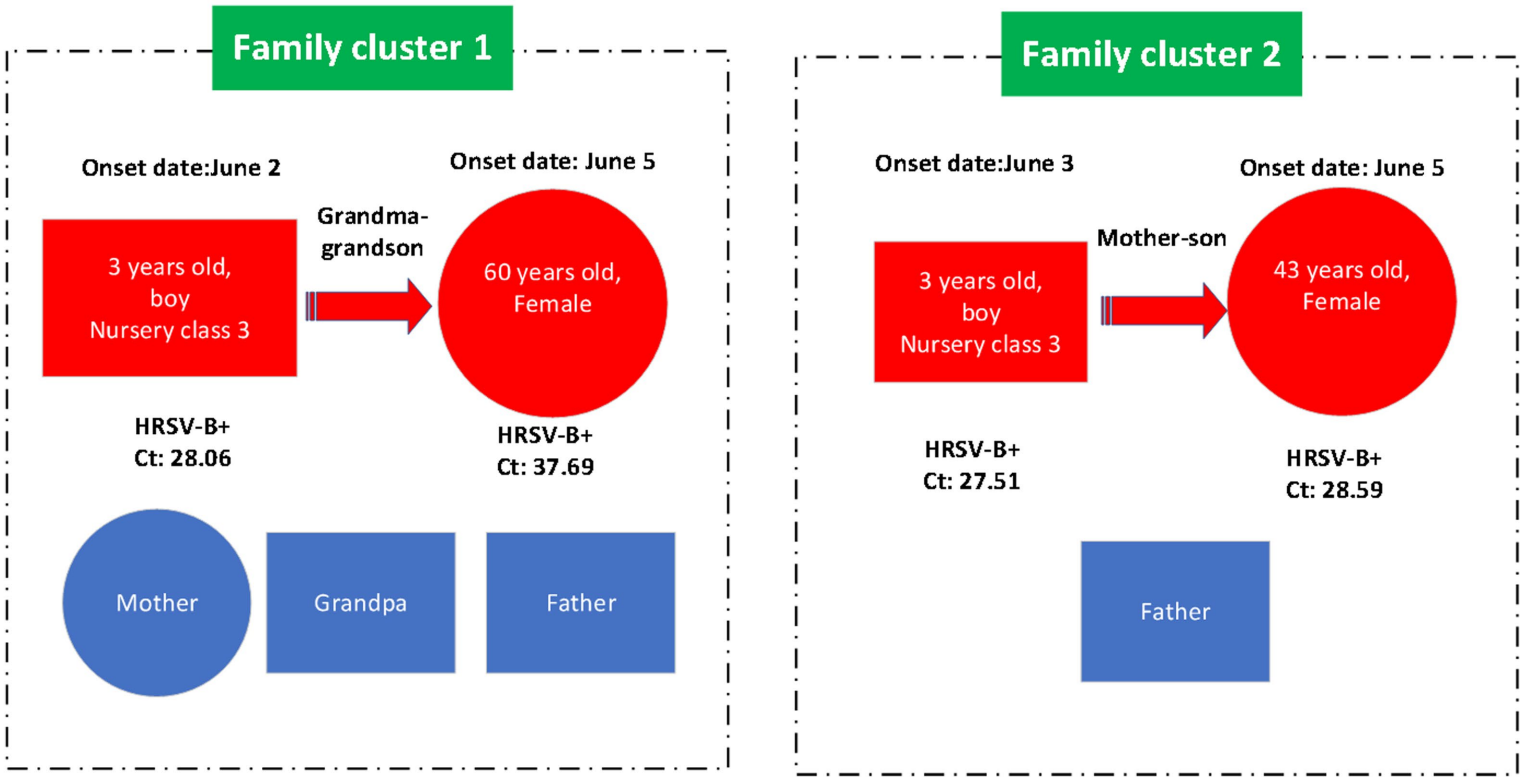
Transmission routes of two-family clusters in 25 families during the human respiratory syncytial virus outbreak in the Boji kindergarten from Daishan countryside, Zhoushan city, Zhejiang Province, China from May to June 2023.

A total of 88 household members (25 families) were investigated. The median number of family members were 3.2 (range: 2–5), and there were two family members with respiratory symptoms during the field investigation. The day from the index case to the secondary case was 3 days. Of the 20 pharyngeal swabs were collected from the household members, 2/20 were HRSV positive; one was a grandmother (a 60-year-old female) of a boy from nursery class 3 who was HRSV-B positive (Ct value: 37.69); the other was a mother (a 43-year-old female) of a boy from nursery class 3 who was HRSV-B positive (Ct value: 29.58) (Figure 4).

### Nosocomial infection investigation

At the time of investigation, four cases were admitted to different rooms of the paediatrics department of the pediatric ward of Daishan First People’s Hospital. On June 8, one bedside caregiver of the case admitted to bed 16 reported the onset of fever and cough. In response to this event, we conducted a nosocomial infection investigation. A total of 17 throat swabs were collected from 8 bedside caregivers, 2 medical staff, 4 outbreak cases due to HRSV, and 3 sporadic cases due to HRSV. Among these samples, we identified six to be HRSV positive. One bedside caregiver for bed 16 (Ct value = 29.58) had HRSV genotype B, two outbreak cases infected with HRSV genotype B, while two spondaic cases caused by HRSV genotype B. However, the two medical staff members were negative for HRSV.

### Molecular epidemiological characteristics

**Figure 5:**
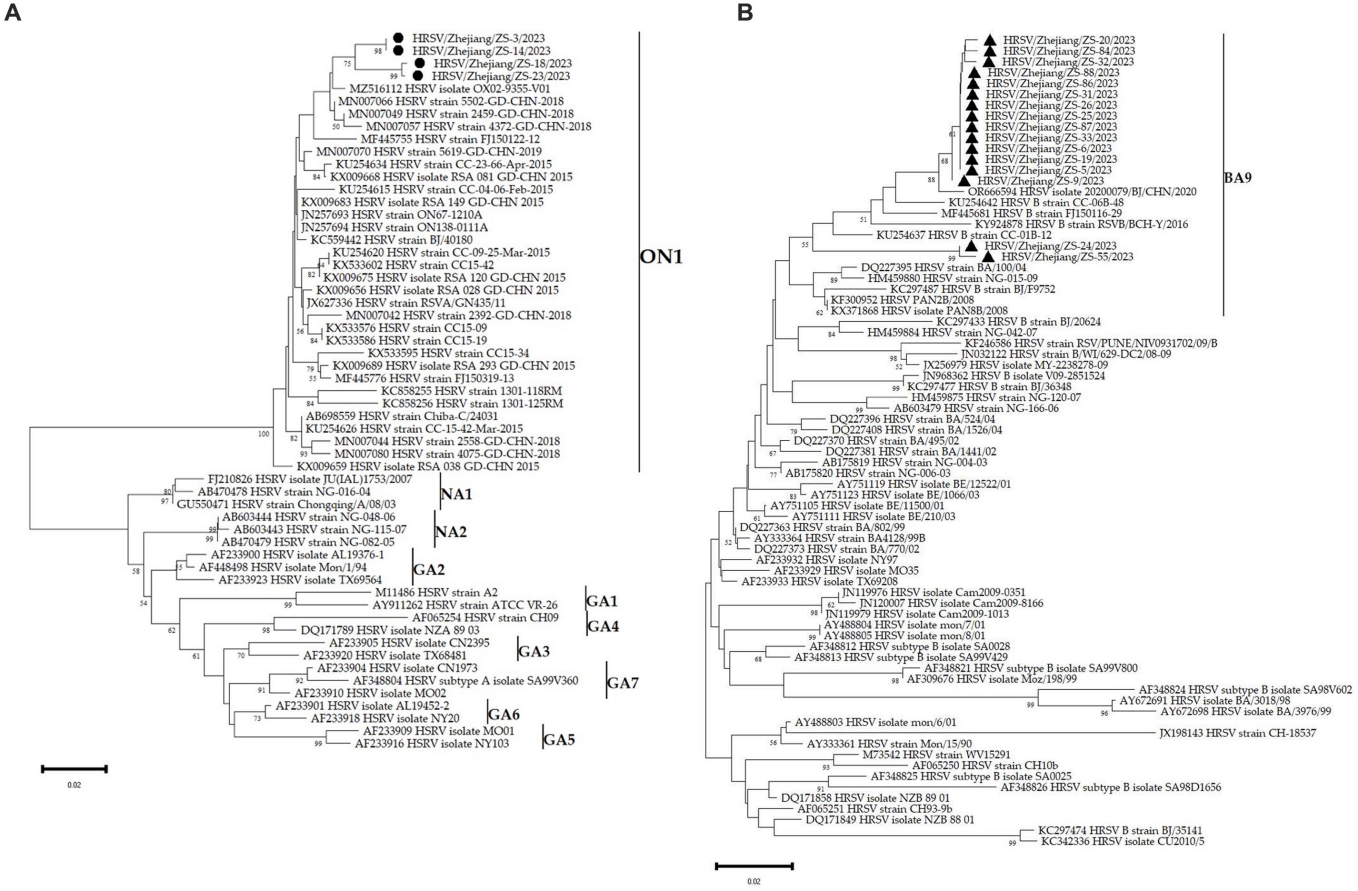
Phylogenetic tree of HVR2 fragment of the G sequences of HRSV. **(A)** The viruses reported in this study are highlighted with circle, including 2 outbreak cases from the class 4 and the other two sporadic cases (HRSV/Zhejiang/ZS3/2023, HRSV/Zhejiang/ZS14/2023) Zhejiang Province, China, from May to June 2023. **(B)** The viruses reported in this study are highlighted with circle, including 16 outbreak cases from the class 2,3,4, Zhejiang Province, China, from May to June 2023. HRSV referenced sequences were downloaded from GenBank. Phylogenetic trees were generated using MEGA with the maximum likelihood method.

The HVR2 fragment of the G gene of HRSV (324 nucleotides) was successfully obtained from 16 outbreak cases and two household members. The sequences from two children in the class 4 were of genotype A, and the other 16 sequences belonged to genotype B. Homology analysis of genotypes A and B revealed that these sequences were 99.99 and 100% identical, respectively. Phylogenetic trees were constructed using the 18 sequences from this study and HRSV B reference sequences downloaded from the GenBank database. The 18 strains clustered into the same branch as the B/BA9 genotype reference sequences. However, the remaining two sequences were clustered to the same branch as the A/ON1 genotype reference sequences. The same results were obtained by performing a BLAST search of the Daishan sequences against the GenBank database (see Figures 5A,B). HSV ON1 and BA9 sequences in this outbreak were closely related to contemporary viruses by using complete genome phylogenetic analysis with all historical and recent HRSV sequences in Zhejiang Province related with databases up to December 2023.

## Discussion

This report provides the first description of an outbreak of HRSV/B mixed HRSV/A in a kindergarten in an island area in eastern China. This large outbreak started on May 18 of 2023, resulted in an overall clinical attack rate of 53.19% and a microbiologically confirmed attack rate of nearly 24.3%. A series of comprehensive non-pharmaceutical interventions, especially school/class closure were implemented to end this outbreak on June 15. Furthermore, this HRSV outbreak in the kindergarten linked with two family clusters. There is a strong link between this HSV outbreak and local community transmission.

The effects of COVID-19 pandemic lockdown measures on the HRSV ecosystem have been reported. Before the COVID-19 pandemic, the seasonal nature of HRSV circulation usually followed a predictable pattern, with epidemics occurring from November to April in the Northern Hemisphere (14). However, the COVID-19 pandemic and the introduction of nonpharmaceutical interventions have caused substantial changes in HRSV seasonality and epidemiology (14). Many countries, such as the United States, the United Kingdom, and Austria, have experienced an absence of RSV during the typical season, followed by an out-of-season surge upon relaxation of non-pharmaceutical interventions (14). For example, Sara Manti et al. from Italy reported the experience of the SARS-CoV-2 outbreak has led to a marked decrease in other viral respiratory infections in 2020, such as HRSV, but an increased number of susceptible individuals to HRSV infection in the autumn-winter of 2021 (15). Raffaella Nenna et al. reported that demonstrated an early and intense peak in RSV-associated pediatric admissions in Italy (16). Ujiie et al. from Japan indicated that a substantial outbreak of HRSV infection in Tokyo starting in spring 2021 (17). Like the other countries, the number of HRSV infections began to climb suddenly from March to April in China since non-pharmaceutical interventions were lifted for SARS-CoV-2 at the end of 2022.

Icelandic data highlighted that HRSV infection during the COVID-19 pandemic was more likely to occur in older children, according to a comparison across the five previous seasons (16 months vs. 5.7 months) (14, 18). For example, a study from France reported that the median age of infected children in 2020/2021 was higher than in previous years (15). Anna et al. from Italy reported that comparing the two seasons, age at admission was significantly higher in the current season (median age 2022–2023 65 days vs. median age 2018–2019 58 days).These changes in vulnerable age groups increased the possibility of HRSV outbreaks in kindergarten (18, 19), which is an ideal setting for institutional transmission and amplification of respiratory viruses due to the frequency and intensity of contact between kindergarten children (19). Due to the non-seasonal increase in HRSV prevalence, national multiple respiratory pathogen surveillance network has been strengthened. A large HRSV outbreak was identified in the kindergarten located on the island of Zhejiang Province from May to June of 2023 by this network. The epidemic investigation displayed widespread transmission of the HRSV that occurred in the kindergarten. This observation could be due to several reasons. First and most importantly, this HRSV outbreak was an extension of the community transmission (1/3 of HRSV outbreak cases were clustered in the same community) when HRSV surged in this area (4). Second, the outbreak was not recognized, reported, or responded to during its early phase; the Children’s Day group activities on June 1 must have sped up the propagation of the epidemic. Third, HRSV can survive on fomites (toys, paper tissues, and beds) for 4–7 h, and the period of shedding is one week; longer periods have been observed in young children and immunocompromised patients (1, 20–22). Furthermore, HRSV is a very contagious infectious disease with an R0 of 4.5, much higher than seasonal influenzas (R0 = 1.5–2.0) (23). The attack rate in this study differed substantially by class and age group. The highest attack rate occurred in nursery class 3 (29–42 months), and the lowest was seen in the junior and senior classes (59–88 months). The age of children from the nursery classes ranged from 29 to 43 months, and they had increased susceptibility to HRSV infection due to a so-called immunity debt (24). Fortunately, all patients made a full recovery, and there were no deaths.

Despite the increased circulation of respiratory viruses during the COVID-19 pandemic, some study did not reveal significant differences in the severity of respiratory infections between the current and previous epidemics (25). On the contrast, a few of studies have indicated that the effects of the delayed HRSV season might increase severe HRSV-related diseases in infants (26, 27). Such as, a study from France indicated that delayed HRSV outbreak was associated with more hospitalizations for ALRI, higher age of pediatric inpatients. A similar outcome was also observed in this study, with a higher percentage of previously healthy children presenting with fever, one-third hospitalization, and longer hospital stay (28, 29). In this outbreak, no critical cases were identified in these admitted cases. No one needs oxygen therapy and noninvasive positive pressure ventilation. Increased severity of this kindergarten related HRSV outbreak might have been caused by diminished protective immunity in the population from prolonged low exposure to this virus; delayed diagnosis and treatment (The median day from onset to be admitted, from onset to be confirmed was 2.3 days (raged 0–4 days) for each), resulting in high viral loads (Ct value was between 20 and 30). Although there are different views on whether multi-pathogen infections increase disease severity, for example, Brand et al. reported that the Disease severity in children with bronchiolitis is not associated with infection by multiple viruses (30, 31). Another research indicated that the co-infection with influenza A virus causes more severe body weight loss and more severe and prolonged pneumonia in SARS-CoV-2 (32). In this outbreak, coinfection, as the combination of *Mycoplasma pneumonia* and SARS-CoV-2 virus infections is also the cause of increased disease severity (33).

Local public health authorities can take multi-layered approach to help prevent introduction & spread of SARS-CoV-2 in schools (1, 13). They should increase healthcare professionals’ awareness and enhance hospital preparedness for HRSV from May to June. They should implement and improve the surveillance of HRSV and testing for respiratory pathogens (9, 11, 34, 35). Parents should be asked to keep ill children at home, and good hygienic practices and non-pharmaceutical interventions should be implemented in the community and kindergarten (36). Such interventions are inexpensive and easily accessible. School closure is a public health tool for controlling the respiratory virus, and should include influenza pandemic preparedness plans based on high infectiousness and susceptibility in schoolchildren and high contact rates (37). This kindergarten (all classes) was temporarily closed for one week, starting on June 9, 2023, which strongly affected the circulation of HRSV. The outbreak ended on June 15.

Our study has some limitations, not all the members of teaching staff and households have been tested HRSV. It is not clear that how many HRSV asymptomatic infections were found among parents and teachers and their potentially important role in this school outbreak. Patrick K et al. reported that the frequency of symptomless HRSV infection episodes attained to (42.0%) within the households. Factors independently associated with an increased risk of asymptomatic HRSV infection episodes were higher age, shorter duration of infection, bigger household size, lower peak viral load, absence of concurrent HRSV infections (38). Secondly, we cannot find the first case and the accurate source of this HRSV outbreak.

## Conclusion

This study describes two HRSV subgroups, A/ON1 and B/BA9, as the causative agents of a large outbreak in a kindergarten in Zhejiang Province, China in May–June 2023, which parallels outbreaks in the community and family with the possibility of repeated introductions.

There are three distinct features of this outbreak compared with previous seasons: first, the affected group was the nursery classes, with children older than previously reported; second, a higher attack rate indicated widespread transmission of the HRSV occurred in the kindergarten; and third, the HRSV infection was the most serious, with higher fever, higher admission rates, and longer hospital stay. Real-time genomic surveillance of HRSV outbreak did not reveal specific changes in HRSV that would account for increased the disease severity. Our data suggest that HRSV outbreak in the kindergarten is likely because of diminished protective immunity in the population from low RSV exposure, a consequence of pandemic mitigation measures. Our findings highlight when there is widespread community transmission of HRSV or the number of cases is rising, preventive and protective measures in schools or kindergarten are even more important. Particularly, it is very essential to standardize surveillance system for multi-pathogens, covering a wider range of areas and Chinese school-aged children, which contributed to the early detection and control of outbreaks.

After SARS-CoV-2 restrictions mitigation, the co-circulation of respiratory viruses along with SARS-CoV-2 were observed (39). This situation can lead to the changes of the epidemic dynamics, including changes in the age of the targeted population, disease course and severity. All these highlighted the need for prospective epidemiologic studies and mathematical modelling able to predict the timing and magnitude of epidemics caused by the respiratory virus interactions aimed to adjust better public health interventions (40).

## Data availability statement

The raw data supporting the conclusions of this article will be made available by the authors, without undue reservation.

## Ethics statement

The studies involving humans were approved by the fourth session of the Ethics Review Committee of Zhejiang Province Centre for Disease Control and Prevention. The studies were conducted in accordance with the local legislation and institutional requirements. The human samples used in this study were acquired primarily as part of a previous study for which ethical approval was obtained. Written informed consent for participation was not required from the participants or the participants’ legal guardians/next of kin in accordance with the national legislation and institutional requirements. Written informed consent was obtained from the minor(s)’ legal guardian/next of kin for the publication of any potentially identifiable images or data included in this article.

## Author contributions

SL: Conceptualization, Data curation, Formal analysis, Methodology, Software, Writing – original draft. JP: Data curation, Formal analysis, Software, Writing – original draft. YC: Investigation, Supervision, Writing – original draft. LY: Data curation, Formal analysis, Software, Writing – original draft. EC: Data curation, Formal analysis, Software, Writing – original draft. XW: Data curation, Formal analysis, Software, Writing – original draft. WW: Data curation, Formal analysis, Software, Writing – original draft. BW: Investigation, Supervision, Writing – original draft. XQ: Investigation, Supervision, Writing – original draft. T-CC: Investigation, Supervision, Writing – original draft. WS: Investigation, Supervision, Writing – original draft. ZY: Investigation, Supervision, Writing – original draft. TZ: Writing – original draft, Writing – review & editing. JY: Writing – original draft, Writing – review & editing. JJ: Writing – original draft, Writing – review & editing.
