# Supplementary material for: Human respiratory syncytial virus subgroups A and B outbreak in a kindergarten in Zhejiang Province, China, 2023
Source: Front Public Health. 2024 Feb 16;12:1368744. doi: 10.3389/fpubh.2024.1368744 (PMC10904655; doi:10.3389/fpubh.2024.1368744)
Supplement: Supplementary file 1 [file Table_1.DOCX]

**Supplementary files**

[**Human respiratory syncytial virus subgroups A and B outbreak**](https://pubmed.ncbi.nlm.nih.gov/37189878/) **in a kindergarten linked family and community transmission in Zhejiang Province, China, 2023**


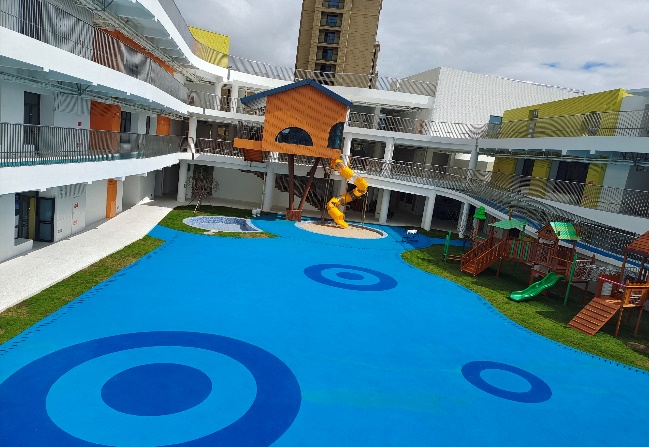

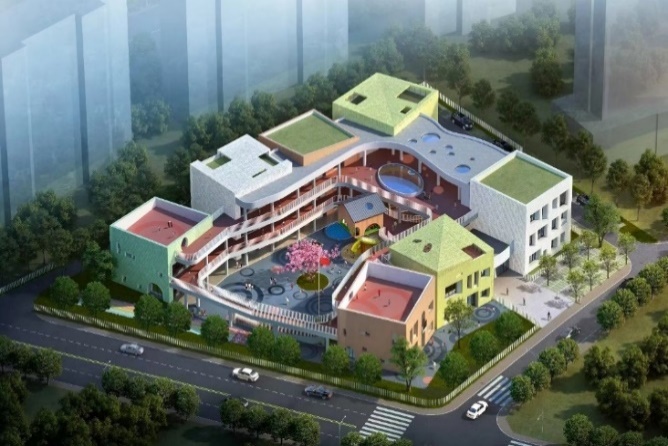


**Supplementary Figure 1**. Architectural spatial distribution of Boji Kindergarten, Daishan countryside, Zhoushan city, Zhejiang Province, China

**Notes:** There are one teaching building, and one playground in this Kindergarten. Teaching building has a total of three floors, four nursery classes are located on the first floor located in the northern building, junior classes and senior classes are located on the second floor of the southern building, and the third floor is idle. Confirmed cases have been reported in Class 3 and Class 4, which are adjacent to each other and located on the first floor of the North Teaching Building. Each classroom is about 130 square meters and is naturally ventilated. The functional areas are clear in each class, including activity area, lunch break area, toilet and teachers' workshop


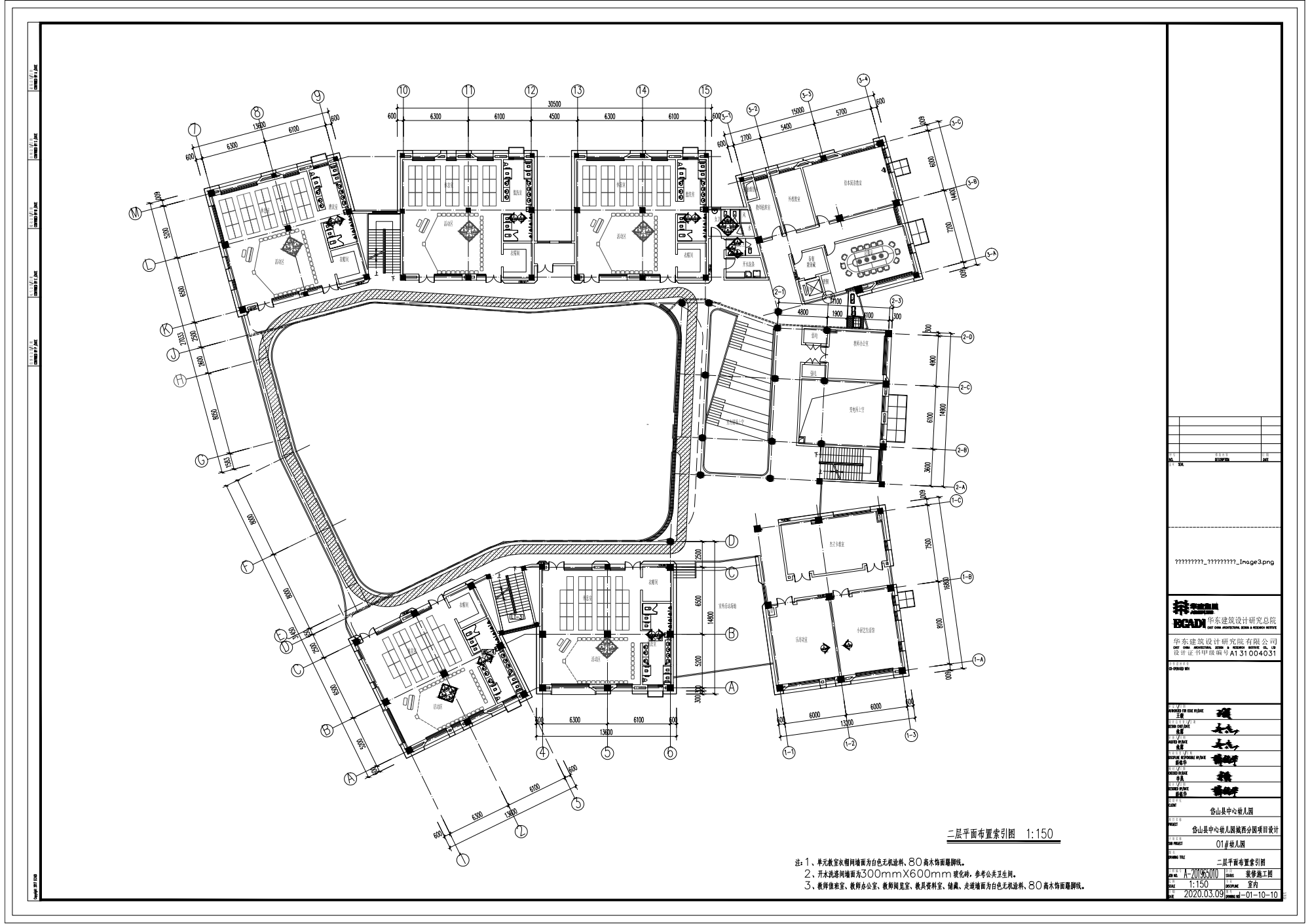


Nursery class 1

Nursery class 2

Nursery class 4

Nursery class 3

**Supplementary Figure 2**. Layout of the first floor of the kindergarten building in the Boji Kindergarten, Daishan countryside, Zhoushan city, Zhejiang Province, China.


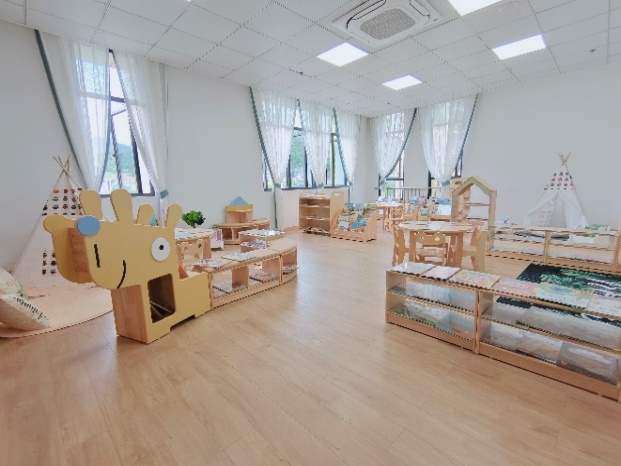

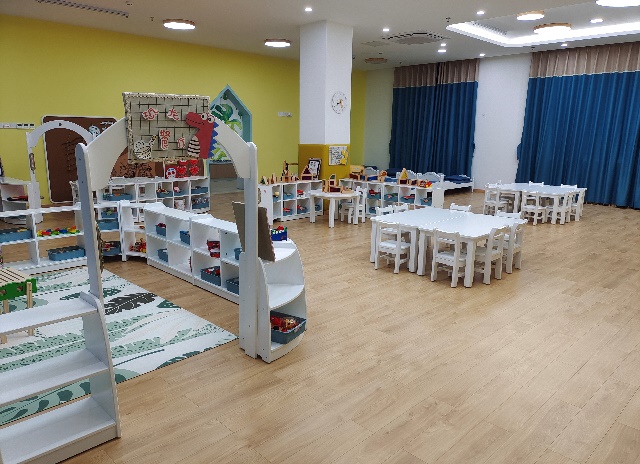


**Supplementary Figure 3**. A realistic picture of Nursery class 3, Boji Kindergarten, Daishan countryside, Zhoushan city, Zhejiang Province, China

 **Supplementary Figure 4.** Diagrammatic sketch of the nursery class 4 with the most affected class in of human respiratory syncytial virus outbreak in Boji kindergarten Daishan countryside, Zhoushan city, Zhejiang Province, China during May to June, 2023

Notes: 24 environmental samples taken from this kindergarten on June 8,2023 were negative for HRSV

**Supplementary Figure 5.** Date of absence among the kids, by class, in the Boji kindergarten from Daishan countryside, Zhoushan city, Zhejiang Province, China from May to June 2023. (n=67)

**Supplementary Table 1.** The attack rates for HRSV by grade/age group and sex in 45 HRSV suspected cases among children in a private kindergarten in Daishan countryside, Zhoushan city, Zhejiang Province, China by date of illness onset, May to June of 2023 (n = 45)

| Category | No. of cases | No. of children | Attack rate (%) | Attack rate ratio (95 % CI) | P value |
| --- | --- | --- | --- | --- | --- |
| Overall attack rate | 45 | 103 | 43.69% |  |  |
| Attack rate by sex |  |  |  |  |  |
| Boys | 25 | 56 | 44.6 | 1.047（0.967，1.133） | 0.255 |
| Girls | 20 | 47 | 42.6 | Reference |  |
| Attack rate by class (age-range in months) |  |  |  |  |  |
| Nursery class 1 | 1 | 18 | 5.6 | 0.112（0.026，0.473） | 0.003 |
| （29~45） |  |  |  |  |  |
| Nursery class 2 | 9 | 18 | 50 | Reference |  |
| （38~43） |  |  |  |  |  |
| Nursery class 3 | 17 | 19 | 89.5 | 1.790（1.159，2.764） | 0.009 |
| （29~42） |  |  |  |  |  |
| Nursery class 4 | 16 | 18 | 88.9 | 1.778（1.139，2.776） | 0.011 |
| （23~43） |  |  |  |  |  |
| Junior class | 2 | 18 | 11.1 | 0.222（0.069，0.712） | 0.011 |
| （42~56） |  |  |  |  |  |
| Senior class | 0 | 12 | 0 | 0 | 0.003 |
| （59~88） |  |  |  |  |  |

**Supplementary Table 2.** Characteristics of 18 non-admitted cases of human respiratory syncytial virus infection in the children from Boji kindergarten Daishan countryside, Zhoushan city, Zhejiang Province, China from May to June 2023

| No. of case | Class name | Age  (years) | Gender | Sampling date | HRSV  (Ct value) | HRSV genotypes | Sars-CoV-2 | Influenza A | Influenza B | Adenovirus | Rhinovirus |
| --- | --- | --- | --- | --- | --- | --- | --- | --- | --- | --- | --- |
| 1 | Nursery class 2 | 3 | Male | June 9 | Positive  (26.63) | B | Negative | Negative | Negative | Negative | Negative |
| 2 | Nursery class 2 | 3 | Female | June 9 | Positive  (37.79) | B | Negative | Negative | Negative | Negative | Negative |
| 3 | Nursery class 3 | 3 | Male | June 9 | Positive  (33.5) | B | Negative | Negative | Negative | Negative | Negative |
| 4 | Nursery class 3 | 2 | Male | June 9 | Positive  (38.4 ) | Unknown | Negative | Negative | Negative | Negative | Negative |
| 5 | Nursery class 3 | 2 | Female | June 9 | Positive  (30.9 ) | B | Negative | Negative | Negative | Negative | Negative |
| 6 | Nursery class 3 | 3 | Male | June 9 | Positive  (35.8) | B | Negative | Negative | Negative | Negative | Negative |
| 7 | Nursery class 3 | 3 | Female | June 9 | Positive  (28.9 ) | B | Negative | Negative | Negative | Negative | Negative |
| 8 | Nursery class 3 | 3 | Female | June 9 | Positive  (30.24 ) | B | Negative | Negative | Negative | Negative | Negative |
| 9 | Nursery class 3 | 3 | Male | June 9 | Positive  (28.06 ) | B | Negative | Negative | Negative | Negative | Negative |
| 10 | Nursery class 3 | 2 | Female | June 9 | Positive  (30.9) | B | Negative | Negative | Negative | Negative | Negative |
| 11 | Nursery class 3 | 3 | Female | June 9 | Positive  (37.71 ) | Unknown | Negative | Negative | Negative | Negative | Negative |
| 12 | Nursery class 3 | 3 | Male | June 9 | Positive  (26.42) | B | Negative | Negative | Negative | Negative | Negative |
| 13 | Nursery class 3 | 3 | Male | June 9 | Positive  (39.71) | B | Negative | Negative | Negative | Negative | Negative |
| 14 | Nursery class 3 | 2 | Male | June 9 | Positive  (22.98 ) | B | Negative | Negative | Negative | Negative | Negative |
| 15 | Nursery class 4 | 2 | Female | June 9 | Positive  (36.85) | A | Negative | Negative | Negative | Negative | Negative |
| 16 | Nursery class 4 | 2 | Male | June 9 | Positive  (33 ) | A | Negative | Negative | Negative | Negative | Negative |

**Supplementary Table 3.** Symptoms reported by 27 confirmed HRSV outbreak cases among kids and households, in Boji kindergarten Daishan countryside, Zhoushan city, Zhejiang Province, China during May to June, 2023 (n=27 confirmed cases*)

| Symptoms | No. of cases | Percentage (%) |
| --- | --- | --- |
| Fever | 22 | 81.5 |
| Cough | 24 | 88.9 |
| Productive cough | 17 | 63.0 |
| Running nose | 3 | 11.1 |
| Nasal congestion | 2 | 7.4 |
| wheeze | 0 | 0 |
| Sore throat | 0 | 0 |
| Headache | 0 | 0 |
| Sneeze | 0 | 0 |
| Muscle/joint pain | 0 | 0 |
| Vomit | 0 | 0 |
| Diarrhoea | 0 | 0 |
